# The importance of elevated basal 17-hydroxyprogesterone in the diagnosis of children with congenital adrenal hyperplasia

**DOI:** 10.5937/jomb0-54426

**Published:** 2026-03-17

**Authors:** Jelena Miolski, Maja Ješić, Anita Skakić, Sonja Pavlović, Ivana Vorgučin, Vladislav Bojić, Smiljka Kovačević, Jelena Blagojević, Nevena Didić, Mirjana Doknić, Vera Zdravković

**Affiliations:** 1 Children's Department of Neonatology, General Hospital "Stefan Visoki", Smederevska Palanka; 2 University of Belgrade, School of Medicine, Belgrade; 3 University Children's Clinic, Belgrade; 4 Institute of Molecular Genetics and Genetic Engineering, Belgrade; 5 Institute for Health Care of Children and Adolescents of Vojvodina, Department of Endocrinology, Diabetes and Metabolic Diseases, Pediatric Clinic, Novi Sad; 6 University of Novi Sad, Faculty of Medicine, Novi Sad; 7 University Clinical Center of Serbia, Clinic for Endocrinology, Diabetes and Metabolic Diseases, Belgrade

**Keywords:** 17-hydroxyprogesterone, congenital adrenal hyperplasia, precocious puberty, CYP21A2 heterozygous, CYP21A2 gene, 17-hidroksiprogesteron, kongenitalna adrenalna hiperplazija, prevremeni pubertet, CYP21A2 heterozigot, CYP21A2 gen

## Abstract

**Background:**

Congenital adrenal hyperplasia (CAH) is usually characterised by a deficiency of 21a-hydroxylase, which causes a deficiency of cortisol and aldosterone and an overproduction of 17-hydroxyprogesterone. The classic and non-classical forms of the disease present ambiguous genitalia with signs of precocious puberty (PP) with accelerated height velocity and bone age. accelerated body and bone growth. Elevated basal and stimulated 17-hydroxyprogesterone and genetic testing are crucial for confirming a definitive diagnosis. The aim to determine determine the significance of elevated basal 17-hydroxyprogesterone in children with signs of precocious puberty in the final diagnosis of Congenital Adrenal Hyperplasia.

**Methods:**

A prospective study was conducted at the University Children's Clinic and the Institute of Molecular Genetics and Genetic Engineering in Belgrade from 2019 to 2024. The study involved 64 subjects of both sexes, aged up to 18 years, with precocious puberty, accelerated height velocity and bone age and/or elevated basal 17-hydroxyprogesterone, who were divided into two groups based on the presence/absence of pathogenic variants in the CYP21A2 gene. The anthropometric measures, skeletal maturation and hormone levels were compared between those two groups.

**Results:**

The research included 64 subjects, of whom 30 confirmed CAH and 34 were part of the control group with PR A statistically significant difference was shown in basal (p &lt; 0 .0 0 1 ) and stimulated 17-hydroxyprogesterone (p = 0 .0 1 3 ), cortisol (p = 0.015) and androstenedione (p= 0.014) in homozygous carriers of pathogenic variants in the CYP21A2 gene.

**Conclusions:**

Clinical and laboratory parameters such as precocious puberty and 17-hydroxyprogesterone may be significant hints to consider a carrier mutation for congenital adrenal hyperplasia.

## Abstract (sr)

**Uvod:**

Kongenitalna adrenalna hiperplazija (KAH) je oboljenje u kom nedostatak 21a-hidroksilaze uzrokuje deficit kortizola i aldosterona, sa posledičnim nagomilavanjem 17-hidroksiprogesterona. Klasičnu i neklasičnu formu bolesti predstavljaju ambivalentne genitalije sa znacima prevremenog puberteta (PP) puberteta (PP), ubrzanog telesnog i koštanog sazrevanja. Klinički znaci zahtevaju laboratorijsku (povišen bazalni i stimulisani 17-hidroksiprogesteron) i genetičku potvrdu konačne dijagnoze. Cilj studije je utvrditi značaj povišenog bazalnog 17-hidroksiprogesterona kod dece sa znacima prevremenog puberteta u potvrdi dijagnoze kongenitalne adrenalne hiperplazije.

**Metode:**

Prospektivna studija sprovedena je u Univerzitetskoj dečijoj klinici i Institutu za molekularnu genetiku i genetičko inženjerstvo u Beogradu od 2019. do 2024. godine. U studiji je učestvovalo 64 ispitanika oba pola, uzrasta do 18 godina, sa prevremenim pubertetom, ubrzanim koštanim i telesnim rastom, povišenim bazalnim 17-hidroksiprogesteronom, koji su podeljeni u dve grupe na osnovu prisustva/odsustva patogenih varijanti u genu CYP21A2. Obavljen je fizikalni pregled, antropometrijska merenja, procena skeletnog sazrevanja, uzeta krv za biohemijske i genetičku analizu, na osnovu kojih su ove dve grupe ispitanika upoređivane.

**Rezultati:**

Istraživanjem je obuhvaćeno 64 ispitanika, podeljenih na dve grupe, sa potvrđenom dijagnozom KAH (30 ispitanika) i sa PR kao kontrolnom grupom (34 ispitanika). Rokazana je statistički značajna razlika u bazalnom (p &lt; 0 ,0 0 1 ) i stimulisanom 1 7-hidroksiprogesteronu (p= 0,013), kortizolu (p= 0,015) i androstenedionu (p= 0,014) kod homozigotnih nosioca patogenih varijanti u genu CYP21A2.

**Zaključak:**

Klinički i laboratorijski pokazatelji poput prevremenog puberteta i 17-hidroksiprogesterona mogu biti značajni indikatori nosioca varijanti odgovornih za razvoj kongenitalne adrenalne hiperplazije.

## Introduction

Congenital adrenal hyperplasia (CAH) is an inherited disease characterised by biallelic genetic variants in the CYP21A2 gene, causing predominantly (95%) 21a-hydroxylase enzyme deficiency, reducing adrenal steroidogenesis. The incidence is variable in the general population; the non-classical form is more prevalent (0.1%) than the classical form (1:18000) [1][2][3].

The clinical manifestations of the classic form of salt-wasting (SW) or the simple virilising (SV) form are due to a deficiency of cortisol and aldosterone, the accumulation of 17-hydroxyprogesterone (17-OHP), which in steroidogenesis should metabolise the deficient enzyme and the activation of alternative pathways with the formation of excess adrenal androgens [4]. In the neonatal period, the classic form of SW CAH is challenging to diagnose in male neonates who present with failure to thrive, vomiting or electrolyte disturbance, and severe adrenal crisis that may lead to death. The form of SV CAH is characterised by normal secretion of aldosterone with cortisol deficiency, presenting with clinical signs of precocious puberty (PP) in childhood. Boys usually have penile growth and scrotum without enlargement of testicular size, and girls present with premature pubarche with clitoromegaly. In girls with a non-classical form of CAH (NC), in addition to premature pubarche, we notice hirsutism, acne, and menstrual cycle disorders as the consequences of the synthesis of excess androgens. All forms of CAH are characterised by advanced bone maturation due to excess adrenal androgens, causing earlier fusion of bone epiphyses, earlier cessation of growth, and reduced final height [1][5].

## Materials and methods

For the participation of the subjects in the study, the parents signed informed consent forms, which were approved by the Ethics Committee of the Faculty of Medicine University of Belgrade and the Ethics Board of the University Children's Hospital in Belgrade. The study aimed to determine the importance of elevated basal and stimulated 17-hydroxyprogesterone in children with PP height velocity and skeletal maturation in the final diagnosis of CAH.

The prospective study was conducted at the Endocrinology division of the University Children's Hospital and the Institute of Molecular Genetics and Genetic Engineering in Belgrade from January 2019 to April 2024. The study included a total of 64 subjects of both sexes, aged up to 18 years, divided into two groups. The first group consisted of 34 subjects with a genetically confirmed diagnosis, and the second group consisted of 30 subjects with a genetically excluded diagnosis of CAH. Subjects in both groups had clinical characteristics of CAH: virilisation and/or loss of salt or signs of precocious puberty, growth velocity and skeletal maturation, elevated basal values of 17-OHP (>2 ng/mL), lower basal cortisol concentrations compared to the age. Patient data were collected at regular endocrinological visits at intervals of no more than six months and/or at disease evaluation. A physical examination was performed, including height and weight measurement, an X-ray of the left hand and a blood sample was taken for biochemical and genetic analysis. All parents of the patients consented to participate in the study by signing informed consent.

Biochemical measurements included basal and stimulated values of 17-hydroxyprogesterone and cortisol concentrations, as well as basal concentrations of androstenedione and testosterone. The concentration of 17-OHP was measured by the Radioimmunoassay-RIA method in the biochemical laboratory of the Clinical Center of Serbia. A blood sample was taken early in the morning before 8 a.m. and in girls during the early follicular phase of the cycle. Subjects with basal values of 17-OHP>2 ng/mL underwent the ACTH testing [1]. After collecting a basal blood sample, 0.25 mg of the conventional preparation, ACTH-cosyntropin was administered parenterally [6]. After 30 and/or 60 minutes, the concentration of stimulated 17-OHP (s17-OHP) and stimulated cortisol (sC) was determined from the basal blood sample. Aldosterone, testosterone, and cortisol were analysed using a competitive chemiluminescent immunoassay in the biochemical laboratory of the University Children's Clinic in Belgrade. During regular check-ups, an additional blood sample of 5 mL was taken for genetic analyses performed at the Institute of Molecular Genetics and Genetic Engineering in Belgrade. Selective PCR amplification of the active CYP21A2 gene, distinguishing it from the CYP21A1P pseudogene, was performed to specifically detect variants, gene conversions, and large deletions associated with the development of CAH [7].

Anthropometric measurements used standard deviation scores (SDS) calculated using Auxology software version 1.0 b17 (Pfizer, New York, NY, USA) [8]. Body height is measured on an stadiometer, weight with a decimal scale, and the assessment of the degree of nutrition with a body mass index calculated as a body mass quotient expressed in kg and squares of body height in meters. The growth rate was calculated as the difference in body height divided by the time between their measurements. The stages of puberty are defined according to Tanner. Precocious puberty (PP) is defined by the onset of pubarche and/or axillary hairiness before 8 years of age in girls and 9 years of age in boys [9]. Bone maturity was determined after radiography of the left hand, and the acceleration of bone maturation was calculated as the difference between bone and chronological age.

### Statistical analyses

Database creation and data analysis were conducted using the software package for statistics R. The selection of tests corresponds to the content and type of examined characteristics as well as the objectives of the research. For descriptive statistics, measures of central tendency, measures of variability, and graphic and table representation were used. The Mann-Whitney U-test and the Student's T-test were used to test the differences between groups. For a statistically significant value, p<0.05 was taken.

## Results

In a total of 64 subjects, with an age mean of 7.59 and median of 7.75 (range years, the first group with genetically confirmed CAH has 34 subjects; their diagnosis was confirmed at the age of mean 28.89; median 1.00, range months, and the second group has 30 subjects. There is no statistically significant difference between the groups according to gender (F:M, 56:44 vs. 70:30) and age (mean 7.26; median 7.10 vs. mean 7.95; median 7.95 years), but growth velocity values (mean 3.64; median 0.47 vs. mean 10.62; median 2.91 cm) difference was more significant in the group 2 and skeletal maturation (mean 2.52; median 2.19 vs. mean 1.26; median 1.60) was more accelerated in children with CAH, although it did not reach statistical significance (Table 1).

**Table 1:** Comparison of anthropometric characteristics and hormone levels between groups.

Variable	n	CAH				CONTROL			p
		*Mean*	*Median*	*SD*	*n*	*Mean*	*Median*	*SD*	
Age	34	7.26	7.10	5.71	30	7.95	7.95	5.64	0.554
sex M/F	15/19				21/9				
Age at diagnosis	31	28.89	1.00	49.09	N/A	N/A	N/A	N/A	
SDS TV	34	0.94	1.03	1.71	28	0.68	0.81	1.89	0.826
SDS TM	34	0.40	0.26	1.60	28	0.75	1.49	2.04	0.449
SDS BMI	34	-0.09	0.27	1.80	28	0.74	0.91	1.48	0.055
HV SDS	33	3.64	0.47	10.55	28	10.62	2.91	19.91	0.057
BA-CA	20	2.52	2.19	1.64	15	1.26	1.60	1.81	0.051
17OHP (0) (ng/mL)	33	133.68	38.50	369.13	30	9.39	1.99	14.68	<0.001*
17OHP (30) (ng/mL)	9	77.44	49.00	111.84	10	5.40	2.85	5.29	0.013*
17OHP (60) (ng/mL)	9	83.39	50.00	120.94	11	6.47	3.90	5.32	0.013*
Cortisol (0) (μg/dL)	27	9.47	6.54	9.73	25	9.68	8.50	5.52	0.203
Cortisol (30) (μg/dL)	9	12.61	11.27	6.51	15	19.49	19.70	5.71	0.0148*
Cortisol (60) (μg/dL)	9	15.05	11.90	9.24	15	20.53	21.70	6.80	0.189
Androstenedione (ng/mL)	13	12.64	2.54	28.22	6	0.37	0.26	0.36	0.014*
Testosterone (ng/mL)	28	2.54	0.56	6.60	24	1.43	0.21	2.50	0.275

**Figure 1:**
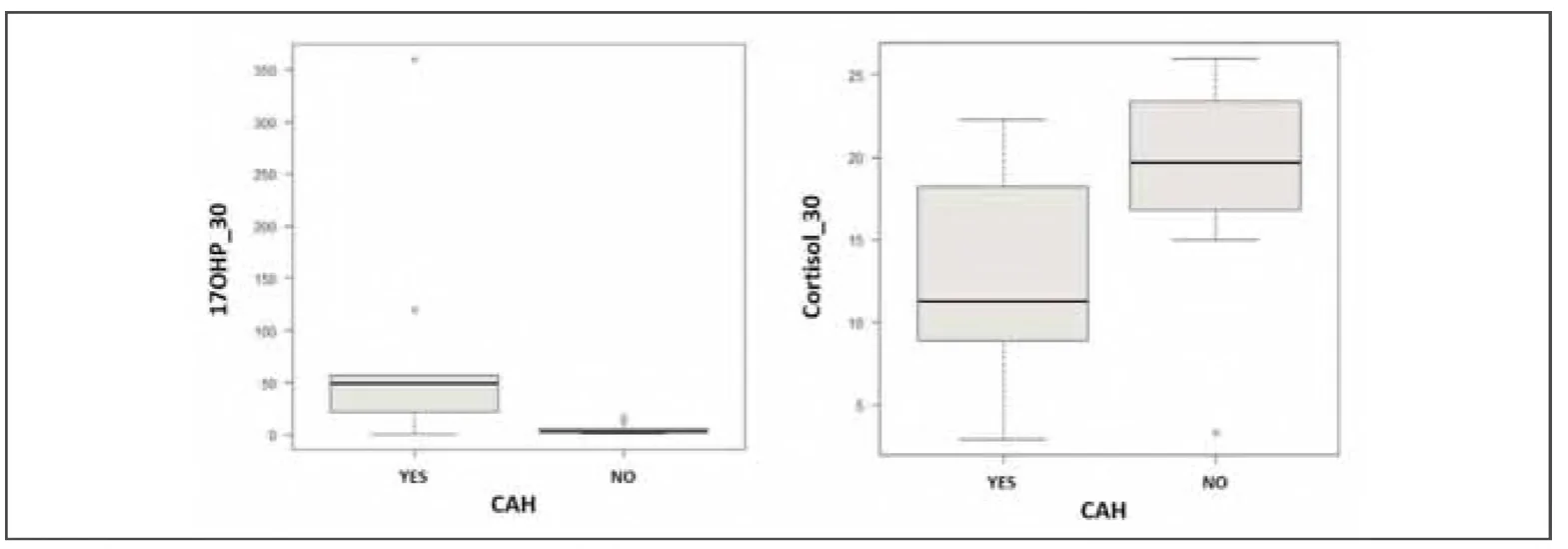
Distribution of s17-OHP 30 and sC 30 between groups.

Basal 17-OHP (b17-OHP) ((mean 133.68; median 38.50 vs. mean 9.39; median 1.99 ng/mL; p< 0.001) was significantly higher in the first group with genetically confirmed CAH. In the first group, the ACTH test was performed in 26% of subjects whose b17-OHP was not clearly elevated or for technical reasons. The remaining subjects (74%) of the first group were diagnosed by genetic analysis based on clinical (PP) and laboratory parameters (b17-OHP>10 ng/mL). One-third of the participants in the second group had undergone ACTH testing because of b17-OHP>2 ng/mL. s17-OHP was significantly higher at the 30th minute ((mean 77.44; median 49.00 vs. mean 5.40; median 2.85 ng/mL; p=0.013) and 60th minute (mean 83.39; median 50.00 vs. mean 6.47; median 3.90 ng/mL; p=0.013) in the first group compared to the second group, Figure 1.

Basal cortisol (mean 9.47; median 6.54 vs. mean 9.68; median 8.50 μg/dL; p=0.203) and sC at 60 minutes (mean 15.05 median 11.90 vs. mean 20.53 median 21.70 μg/dL; p=0.189) do not differ significantly between groups, sC at 30 minutes (mean 12.61 median 11.27 vs. mean 19.49 median 19.70 μg/dL; p=0.015) was considerably higher in the second group, Figure 1. Androstenedione concentrations were significantly higher in the first group (mean 12.64 median 2.54 vs. mean 0.37 median 0.26 ng/mL; p=0.014), and no statistically significant difference in testosterone levels was shown (mean 2.54; median 0.56 vs. mean 1.43; median 0.21 ng/mL; p=0.275), Table 1.

We performed genetic analysis on 66.7% of patients in the control group. The most frequent heterozygous variants in the CYP21A2 gene was p.Val282Leu (40%), and others detected were p.Val281Leu, R356W, p.Arg484Trp, p.Pro483Ser (10%). The mean age at presentation was 9.35 years, with premature pubarche, hirsutism and obesity, as was described in the previous studies [10]. We detected repeated epistaxis (R356W) and depression episodes (I236N, V237E, M239K, V281L, F306+t, Q318X, R356W and P453S), not reported in the literature to the best of our knowledge, Table 2.

**Table 2:** The detected variants and clinical symptoms in the control group. PP - precocious puberty; PCO - polycystic ovary syndrome; SGA - small for gestational age; NA - not available

N	Genetics	Heterozygote variants	Symptoms	Age of onset of<br>symptoms (m)
1	NA		clitoromegaly	2
2	heterozygous I236N, V237E, M239K, V281L, F306+t, Q318X, R356W i P453S	I236N, V237E, M239K, V281L, F306+t, Q318X, R356W i P453S	PP (PCO, depression episode)	70
3	heterozygous c.1450 C>T (p.Arg484Trp)	p.Arg484Trp	PP (pubarcha, accelerated growth)	72
4	NA		hirsutism, acanthosis nigricans, acne, obesity	199
5	heterozygous (c.844G>T, p.Val282Leu)	p.Val282Leu	PP (short stature)	173
6	heterozygous (c.844G>T, p.Val282Leu)	p.Val282Leu	PP (short stature)	196
7	NA		eectrolyte abnormalities	0
8	heterozygous c.844G>T (p.Val282Leu)	p.Val282Leu	PP (pubarcha)	96
9	negative genetics		PCO, obesity, hypertension	167
10	heterozygous c.1447C>T (p.Pro483Ser)	p.Pro483Ser	amenorrhea, obesity, PCO	195
11	heterozygous V281L	V281L	PP (obesity, pubarcha)	55
12	negative genetics		PP (hyperandrogenism, pubarcha)	94
13	negative genetics		PP (pubarcha)	96
14	negative genetics		clitoromegaly	0
15	heterozygous carrier of a large gene conversion up to the 3rd exon	conversions to exon 3	PP(pubarcha)	47
16	NA		PP (pubarcha, hyperandrogenism, accelerated growth)	72
17	NA		ambiguous genitalia	0
18	NA		clitoromegaly	24
19	NA		PP (pubarcha)	2
20	NA		hypertrichosis	63
21	heterozygous R356W	R356W	obesity, epistaxis, heart murmur	110
22	negative genetics		PP (pubarcha)	66
23	negative genetics		PP (pubarcha, thelarch, adrenarche, headache)	78
24	NA		head hypotonia, hydrocele, umbilical hernia, complex heart defect	3
25	negative genetics		PP (thelarche, adrenarche, pubarcha)	89
26	negative genetics		ambiguous genitalia, undescended testicles, anus atresia, heart defects, hypotonia, nystagmus, strabismus	0
27	heterozygous c.844G>T(p.Val282 Leu)	p.Val282Leu	PP (thelarche, obesity, hypertrihosis, advanced bone age)	108
28	negative genetics		clitoromegaly	0
29	negative genetics		electrolyte abnormalities, positive family history	0
30	NA		SGA, adrenarha, short stature, PCO	72

## Discussion

Classical CAH, due to severe enzyme deficits and accumulation of precursors, is characterised by high basal and stimulated 17-OHP Values up to 2 ng/mL are physiological; from 2-10 ng/mL, it is recommended to perform an ACTH test, and >10 ng/mL indicates the diagnosis of CAH [1]. In this study, we analysed a group of patients who presented with signs of precocious puberty. We performed the clinical exam, bone age, 17 OH progesterone and ACTH test, but the final diagnosis of late-onset congenital adrenal hyperplasia was confirmed by genetic testing. In the group of subjects with genetically confirmed CAH, three of them had a basal 17-OHP<2 ng/mL and a normal ACTH test (s17-OHP sC). Clinical manifestations (PP), as well as concentrations of b17-OHP and s17-OHP could be an important indicator and predictor in the diagnosis of CAH. Still, genetic analysis was necessary in our three subjects from the first group in order to avoid the failure of adequate diagnostics and timely therapy.

In the control group, 30% of subjects are carriers of heterozygous variants in the CYP21A2 gene, which is consistent with other published follow-up studies in patients with precocious puberty [10]. Heterozygotes had an average b17-OHP of mean 20.92; median 20.13 ng/mL, and in the majority (80%) >2 ng/mL. Heterozygous carriers for specific variants in the CYP21A2 gene affect the inactivity of the enzyme 21α-hydroxylase, thereby causing specific symptoms. This explains their clinical picture of PP, hyperandrogenism, growth and bone age acceleration [10]. In the control group among subjects with b17-OHP>2 ng/mL (14/30), 57% were heterozygotes, and in subjects with b17-OHP>10 ng/mL (7/34), 72% were heterozygotes, i.e. the higher the concentration of b17OHP the frequency of heterozygous carriers increased.

To avoid false positive 17-OHP the analysis of concentrations depends on the time of daily sampling and the stage of puberty. Activation of the hypothalamic-pituitary axis with pulse secretion of gonadotropins at night requires early morning sampling, pursued among our subjects [1].

Children with negative CAH genetics demonstrated adequately high levels of cortisol (>18 μg/mL) after ACTH stimulation, in contrast to children with positive CAH genetics, who had statistically significantly lower values of stimulated cortisol at 30 minutes due to an inadequate adrenal response [6]. Sources of elevated 17-OHP values could be the gonads or cysts in Polycystic Ovary Syndrome (PCOS) that need confirmation by additional tests [11]. Children with signs of PP and elevated androstenedione also require measurement of 17-OHF? Studies have shown, similarly to our research, higher levels of androstenedione, but not testosterone, in patients with CAH compared to PP [12].

During the study period, we treated 4 female newborns with signs of virilisation, two with electrolyte disorder, one with a positive family history of CAH, and three of whom had a b17-OHP>2 ng/mL. We ruled out the possibility of falsely elevated b17-OHP due to early blood sampling (48h), prematurity, low birth weight or disease. The final confirmation of the diagnosis of CAH in two newborns, and in order to avoid their additional stress, instead of performing the ACTH test, was completed by genetic analyses for technical reasons [13]. The unavailability of neonatal screening and the lack of recognition of clinical manifestations of CAH of a newborn with high b17-OHP halt the risk of missing a timely diagnosis of CAH. The purpose of diagnosing CAH in newborns is the prevention of adrenal crisis, adequate gender recognition and the detection of milder forms of CAH for timely treatment.

## Conclusion

The analysis of 17-OHP in children with PP is essential for adequate and timely diagnosis of CAH. In a group of patients with signs of precocious puberty, by analysing the basal and stimulated 17-OHF? we have identified patients with CAH but also heterozygous carriers of 21 OH variants. Although we do not have specific cut-off values for 17-OHP we suggest that elevated values could predict heterozygous carriers, requiring early detection and further monitoring.

## Dodatak

### Conflict of interest statement

All the authors declare that they have no conflict of interest in this work.
